# Harnessing diet to modulate inflammation and symptom progression in endometriosis

**DOI:** 10.3389/fnut.2026.1776512

**Published:** 2026-08-07

**Authors:** Tyler Thompson, Valerie McAllister, Amanda Strong, Stanley Andrisse

**Affiliations:** 1 Department of Physiology, Howard University College of Medicine, Washington, DC, United States; 2 Department of Physiology and Biophysics, Howard University College of Medicine, Washington, DC, United States

**Keywords:** diet, endometriosis, estrogen, inflammation, primary care

## Abstract

Endometriosis (EMS) is an estrogen-dependent inflammatory disease affecting 10% of reproductive-age women, with up to 50% experiencing infertility. As primary care providers (PCPs) are often the first point of care, understanding dietary factors that influence EMS is crucial. This integrative review identifies dietary components that can modulate the inflammatory environment in EMS. Polyphenols like quercetin, apigenin, and curcumin demonstrated anti-inflammatory and anti-angiogenic effects by inhibiting key mediators like prostaglandins, cytokines, and matrix metalloproteinases. Phytoestrogens, vitamins (A, C, D, E), and minerals (magnesium, selenium, zinc) also exhibited protective effects by reducing oxidative stress and modulating immune function. Conversely, omega-6 fatty acids, trans fats, high glycemic index foods, and red meat consumption were associated with increased inflammation and EMS risk. Increasing intake of fruits, vegetables, omega-3 fatty acids, and dairy products may help alleviate EMS symptoms and progression. These dietary insights can empower PCPs to provide holistic, preventive care for EMS patients, even in areas that lack specialized resources.

## Introduction

Endometriosis (EMS) is an estrogen-dependent disease in women of reproductive age, with features of chronic inflammation. This disease stems from the aberrant growth of uterine tissue beyond the confines of the uterus (1). EMS most commonly involves the ovaries, fallopian tubes, and tissue lining the pelvic cavity; there have been cases of endometrial tissue reaching the lungs, heart, and central nervous system (2). Neighboring tissue can become irritated, eventually developing scar tissue and adhesions—bands of fibrous tissue that can cause pelvic tissues and organs to stick together. Approximately 10% of reproductive-age women are affected by EMS, and among women experiencing infertility, endometriosis is estimated to be present in up to 50% of cases (3). EMS commonly manifests as pelvic pain, often associated with menstrual periods. Other symptoms include: dysmenorrhea (often felt in the lower back and abdomen), pain during intercourse, bowel movements, or urination, excessive vaginal bleeding; and gastrointestinal symptoms such as diarrhea, constipation, bloating, and nausea (1). Because the symptomatology of endometriosis is so varied, it can take an average 8–10 years to diagnose correctly (4).

Although the exact pathophysiology of endometriosis is not yet certain, there are several theories regarding the origin of the ectopic endometrial tissue. Sampson postulated that endometriosis is caused by retrograde menstruation, where menstrual blood containing endometrial cells flows back through the fallopian tubes and into the pelvic cavity instead of out the body. These endometrial cells stick to the walls and surfaces of pelvic organs, where they grow and continue to thicken and bleed over the course of each menstrual cycle (5). Another possible theory is metaplasia of the coelomic epithelium, which is the mesothelial precursor to the female reproductive tract (6). Progesterone and estrogen imbalance are also thought to play a role in the pathogenesis of EMS as cells become resistant to the dampening effects of progesterone and continue to accumulate due to the unopposed effect of estrogen (7). EMS could also be explained by dysfunction of the immune cells that are responsible for recognizing and destroying endometrial-like tissue that is aberrantly growing outside the uterus (8).

The etiology of EMS has a multidimensional landscape. Among the existing knowledge, extensive research has elucidated the heightened production of pro-inflammatory factors. In essence, the presence of this inflammatory basis suggests that EMS shares characteristics with immune-mediated disorders, which subsequently leads to endothelial dysfunction. The primary inflammatory mediators include prostaglandins (PG), interleukins (IL), vascular endothelial growth factor (VEGF), and tumor necrosis factor (TNF), with the nuclear factor-𝜅B (NF-𝜅B) pathway being the principal pathway involved (9). Nutrition and nutritional status may play a role in the etiology of EMS by altering pro-inflammatory and anti-inflammatory components within the local environment, which in turn influence the state of disease.

A diagnosis of endometriosis can only be confirmed through laparoscopic exploration and biopsy of any suspicious tissue. Laparoscopy is also used to determine the location, extent, and size of the endometrial growths, which can be used to determine the staging of the disease. There are several methods of staging EMS, including the American Society of Reproductive Medicine classification and the American Association of Gynecological Laparoscopists classification. Criteria for these classification systems incorporate the extent of the spread of the tissue, the involvement of pelvic structures in the disease, the extent of pelvic adhesions, and the blockage of the fallopian tubes. The stage of endometriosis does not necessarily reflect the level of pain experienced, risk of infertility, or symptoms present, which has become an area of critique for many (10).

Pain severity in EMS is influenced not only by lesion size or disease stage, but also by lesion location, depth of infiltration, neuroangiogenesis, and the magnitude of local inflammatory signaling. Even minimal or superficial disease may produce substantial pelvic pain when lesions involve highly innervated structures or stimulate elevated production of prostaglandins, cytokines, and nerve growth factors. In contrast, infertility is more commonly associated with advanced disease characterized by pelvic adhesions, distorted pelvic anatomy, ovarian endometriomas, and impaired tubal function, although infertility may also occur in earlier stages due to inflammatory alterations in oocyte quality, implantation, and endometrial receptivity (11).

Treatment of EMS is aimed at reducing pain, promoting fertility, and improving quality of life (12). The most used medical treatments for symptomatic EMS include nonsteroidal anti-inflammatory drugs (NSAIDs), oral contraceptive pills (OCPs), hormone therapy, such as gonadotropin-releasing hormone (GnRH) agonists or antagonists, aromatase inhibitors, and laparoscopic removal of the displaced endometrial tissue. The chosen treatment depends on the severity of symptoms in each patient and whether the benefits outweigh the harm. Although complete surgical excision may substantially reduce recurrence risk in some patients, recurrence of symptoms and lesions remains clinically documented depending on disease extent, residual disease burden, hormonal influences, and duration of follow-up. Consequently, long-term symptom management and recurrence prevention remain important clinical goals (13).

Though this disease is gynecological by nature, the majority of affected women seek their primary care provider (PCP) as the initial point of therapeutic intervention and establish this provider as their central source of care throughout the patient’s life course, even with the involvement of other specialty services (3). These PCPs are equipped to make the clinical diagnosis of EMS, begin first-line management, and maintain a long-term plan for this chronic condition (14). In the United States, medical insurance may cover a limited number of yearly appointments with a registered dietitian for preventive nutritional counseling. However, sustaining ongoing and specialized dietary management can pose challenges (15). PCPs hold a distinct opportunity to deliver holistic and preventive care to diverse patient populations, irrespective of access to specialized resources. Diets rich in processed foods, saturated fats, and refined carbohydrates have been associated with increased inflammatory markers and estrogen activity. Whereas anti-inflammatory dietary components such as omega-3 fatty acids, fiber, antioxidants, and phytonutrients may decrease inflammatory signaling and lesion progression. These anti-inflammatory components can be found within many berries, leafy greens, and dairy products. With this knowledge, further exploration of these adjustable dietary factors, which could eventually lead to the formulation of dietary guidelines, holds the potential to enhance the information available to PCPs. In turn, this knowledge can empower patients affected by endometriosis with accessible tools to alleviate symptoms and impede the progression of their condition.

## Purpose

This integrative review seeks to identify various food items and nutrients capable of influencing the inflammatory environment linked to endometriosis. Such insights may be used to inform the formulation of dietary recommendations by PCPs for their patients.

## Methodology

A systematic search utilized electronic retrieval of biomedical research via the National Health Institute’s PubMed database. These databases were searched from June 2022 to June 2023. Articles that included a focus on other disease processes (i.e., Inflammatory Bowel Disease, Irritable Bowel Syndrome, etc.) were removed. Duplicates and articles that were not in English were also removed. Articles with a primary focus on human or *in vivo* studies were prioritized over those utilizing rodent or *in vitro* studies. Extracted studies were categorized according to major mechanistic themes, including inflammatory, endocrine, metabolic, and general pathophysiologic pathways. While some studies with an exclusive primary focus on hormonal dietary interventions were reserved for a complementary project, studies addressing the interplay between endocrine and immune signaling were retained because hormonal and inflammatory mechanisms in endometriosis are closely interconnected and biologically inseparable. Some articles were included in the reference list of previously selected studies. A total of 35 studies were thoroughly reviewed and annotated. Of the 35 full-text articles reviewed, 8 were excluded due to irrelevance, insufficient focus on dietary inflammation, or prioritization for a separate hormone-focused project. Two additional sources were subsequently incorporated, including one USDA Agricultural Research Service (ARS) dietary database resource and one additional clinical study, resulting in 29 studies included in the final qualitative synthesis.

### PubMed

The data range was filtered for studies published between 2009 and 2024 (past 15 years), sorted by “best match.” The following primary search terms were used: “endometriosis, diet” (yielding 106 results), “endometriosis, dietary factors” (yielding 60 results), and “diet effect on endometriosis” (yielding 62 results). The following secondary search terms were used: “endometriosis, inflammation” (yielding 789 results). Although the specific term “estrogenic dominance” was not used as an isolated search term due to inconsistency in its scientific and clinical usage, broader endocrine-related concepts—including estrogen signaling, estrogen metabolism, hormonal regulation, and estrogen-dependent mechanisms—were incorporated throughout the search and study selection framework to capture relevant literature addressing hormonal contributions to endometriosis pathophysiology. Lastly, these were the following tertiary search terms: “endometriosis, nutrition” (yielding 106 results).

Because of substantial heterogeneity in study design and outcome measures, formal meta-analysis and standardized risk-of-bias scoring were not feasible; however, studies were qualitatively evaluated based on methodological rigor, translational relevance, and consistency with existing literature.

### Inclusion criteria for articles reviewed

Of the full-text articles reviewed (*n* = 35), eight were excluded due to irrelevance to dietary inflammation, exclusive focus on hormonal mechanisms, or insufficient methodological rigor, resulting in 29 studies included in the final qualitative synthesis.

**Table:** 

Search strategy	Records identified
Endometriosis	9,232
Endometriosis AND diet	106
Endometriosis AND dietary factors	60
Diet effect on endometriosis	62
Endometriosis AND inflammation	789
Endometriosis AND nutrition	106
Total records identified (overlapping searches)	10,355
Full-text articles reviewed	35
Articles excluded (irrelevant, duplicates, non-dietary focus)	8
Articles included in final synthesis	29

### Review design and rationale

This article was conducted as an integrative review to synthesize evidence across multiple study designs examining the relationship between diet, inflammation, and endometriosis (EMS). An integrative review methodology was selected because the existing literature in this area is highly heterogeneous, encompassing observational epidemiologic studies, randomized and non-randomized clinical trials, animal models, and *in vitro* mechanistic investigations. While systematic reviews and meta-analyses are well suited for narrowly defined clinical questions with homogeneous study designs and standardized outcomes, such approaches were not feasible for the present topic due to substantial variability in dietary exposures, outcome measures, study populations, and experimental models.

The integrative review framework allowed for the inclusion and critical appraisal of diverse forms of evidence to provide a comprehensive understanding of how dietary components may influence molecular pathways implicated in EMS, including inflammatory signaling, oxidative stress, angiogenesis, and estrogen-dependent mechanisms. This approach is particularly appropriate for translational and hypothesis-generating fields, where mechanistic insights from preclinical models must be contextualized alongside emerging clinical and epidemiologic data.

Given the heterogeneity of study designs, populations, interventions, and outcome measures, quantitative synthesis and meta-analysis were not performed, as pooling of results would not have yielded methodologically sound or clinically meaningful estimates. Instead, findings were narratively synthesized and organized by mechanistic and dietary categories to highlight convergent biological themes and clinically relevant patterns.

**Figure 1:**
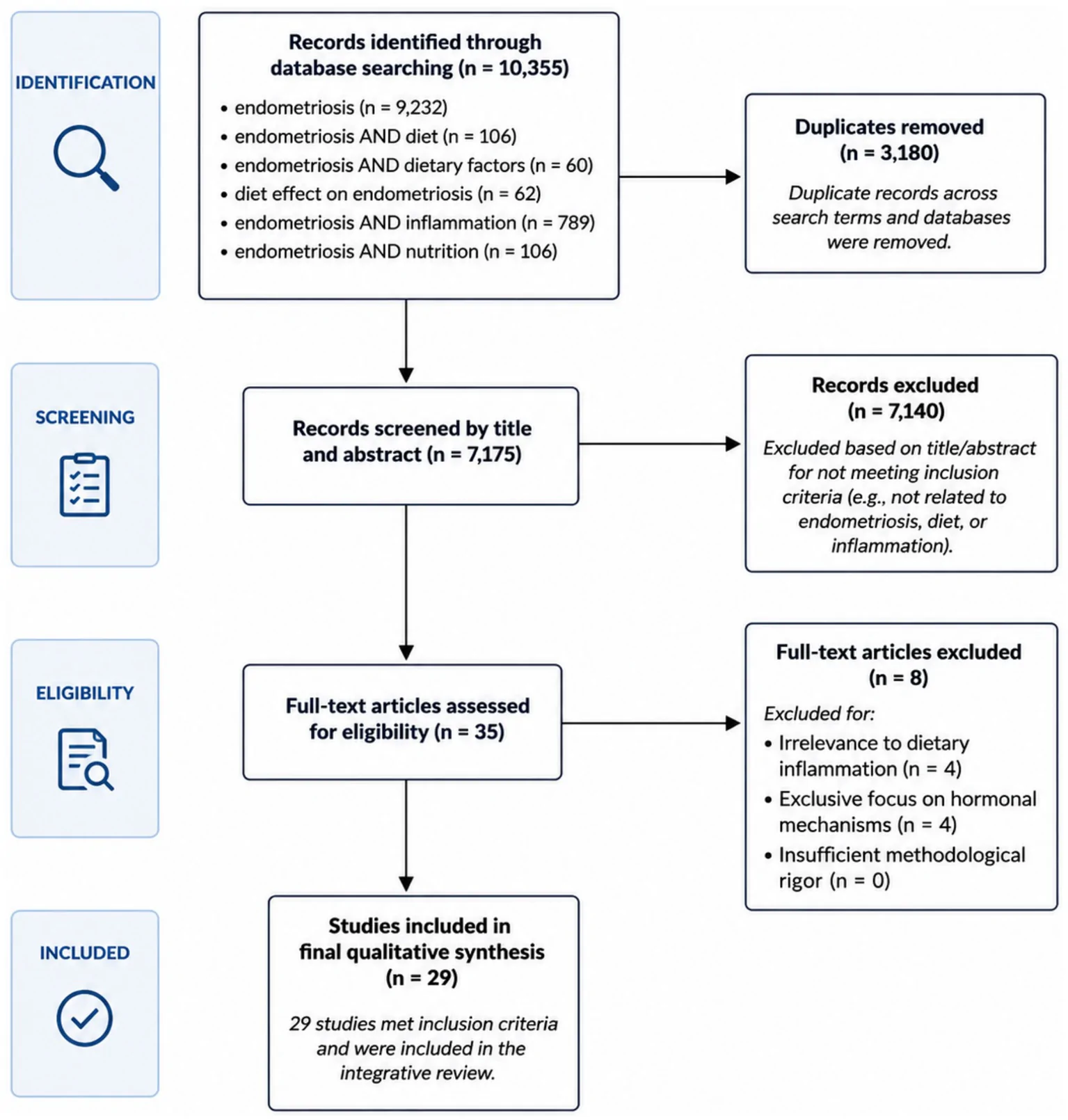
PRISMA-style flow diagram of literature identification, screening, eligibility assessment, and study inclusion. Records were identified through PubMed searches using predefined dietary and inflammation-related search terms. After screening and eligibility assessment, 29 studies were included in the final qualitative synthesis.

A simplified PRISMA-style flow diagram was used to transparently document the literature search and selection process, including database identification, screening, eligibility assessment, and final inclusion of studies, in accordance with best practices for narrative and integrative reviews. The study selection process is summarized in Figure 1.

## Results

### Molecular targets of dietary management

Dietary modification may influence the progression and symptom severity of endometriosis (EMS) through effects on established molecular pathways, including apoptosis resistance, tissue invasion, angiogenesis, immune dysregulation, and oxidative stress (9, 16). Framing these mechanisms clinically highlights the role of nutrition as an adjunct to standard medical management rather than a standalone therapy.

Anti-apoptotic cell survival: Ectopic endometrial tissue exhibits increased expression of estrogen receptor-*β* (ER-β) relative to eutopic endometrium, contributing to immune evasion and resistance to tumor necrosis factor-*α* (TNF-α)–mediated apoptosis (9). Concurrent dysregulation of apoptosis-related proteins—including BCL-2, BCL-XL, BAX, FAS/FASL, caspases, and survivin—further promotes lesion persistence and reduced programmed cell death.

Invasion and implantation: Proinflammatory cytokines, particularly interleukin-1 (IL-1) and interleukin-6 (IL-6), regulate matrix metalloproteinase (MMP) activity. Increased MMP expression facilitates extracellular matrix degradation, enabling ectopic endometrial cells to invade surrounding tissue and establish pelvic implants (9).

Angiogenesis and immune dysregulation: Elevated concentrations of pro-angiogenic mediators are consistently identified in the peritoneal fluid of patients with EMS. These include fibroblast growth factors (FGFs), platelet-derived growth factor (PDGF), transforming growth factors-*α* and -*β* (TGF-α, TGF-β), hepatocyte growth factor (HGF), erythropoietin, angiogenin, TNF-α, and IL-8 (9). Immune dysregulation further contributes to disease activity, as increased peritoneal macrophages secrete inflammatory mediators that impair natural killer cell cytotoxic function. Although macrophages normally participate in immune surveillance and clearance of aberrant endometrial cells, the peritoneal macrophages observed in EMS appear functionally dysregulated. Rather than efficiently eliminating ectopic tissue, these macrophages adopt a predominantly pro-inflammatory phenotype characterized by increased cytokine and growth factor secretion, which may further promote lesion survival, angiogenesis, and local immune tolerance. Concurrently, reduced natural killer (NK) cell cytotoxic activity limits effective clearance of ectopic endometrial cells, allowing persistent implantation despite heightened inflammatory cell presence. Key mediators implicated in this process include IL-1, IL-6, IL-8, TGF-*β*, TNF-*α*, vascular endothelial growth factor (VEGF), cyclooxygenase-2 (COX-2), and monocyte chemoattractant protein-1 (MCP-1).

Oxidative stress: Estrogen-induced upregulation of antioxidant enzymes, including superoxide dismutase (SOD), may paradoxically support ectopic cell survival. Elevated oxidative stress markers observed in serum and peritoneal fluid are associated with inflammation, angiogenesis, and lesion maintenance in EMS (9, 17). Taken together, these pathways represent clinically relevant targets through which dietary interventions may modulate disease activity and symptom expression.

These interconnected inflammatory, angiogenic, oxidative, and immune-mediated pathways provide the mechanistic basis through which specific dietary compounds may influence lesion progression, symptom severity, and overall disease activity in EMS.

### Polyphenols

Polyphenols demonstrate consistent anti-inflammatory, anti-angiogenic, and pro-apoptotic effects in experimental and clinical EMS models. Quercetin has been shown to reduce serum prostaglandin E2 (PGE2), corresponding with decreased inflammatory activity, pain severity, and lesion size (16). Apigenin increases reactive oxygen species (ROS) and endoplasmic reticulum stress, resulting in reduced cellular proliferation and increased apoptosis. These effects are accompanied by downregulation of COX-2, PGE2, and IL-8, as well as inhibition of nuclear factor-κB (NF-κB) signaling (9).

Several polyphenols also target angiogenesis and tissue invasion. Apigenin reduces peritoneal VEGF, TNF-*α*, and IL-6 levels, thereby limiting neovascularization and implant volume. Baicalein suppresses MMP expression and reduces invasive potential. Genistein and daidzein demonstrate synergistic inhibition of NF-κB signaling with subsequent reductions in IL-6, IL-8, COX-2, and PGE2 (9). Puerarin and naringenin further suppress MMP-2, MMP-9, VEGF, TNF-*α*, and anti-apoptotic proteins such as BCL-2, while promoting apoptotic signaling.

Preclinical studies demonstrate that curcumin exhibits broad mechanistic activity, including inhibition of NF-κB, suppression of MMPs, TNF-*α*, VEGF, COX-2, IL-1, IL-6, and IL-8, and enhancement of apoptosis through downregulation of BCL-2 and MMP-9 (9). Combination therapy with curcumin and quercetin has been associated with reduced serum PGE2 and clinically meaningful symptom improvement (16).

Primarily in preclinical models, resveratrol affects multiple disease pathways by decreasing peritoneal VEGF, insulin-like growth factor-1 (IGF-1), IL-6, IL-8, COX-2, TNF-*α*, and MMP activity, while increasing SOD and pro-apoptotic BAX expression. These effects suppress invasion, angiogenesis, and inflammation while promoting apoptosis (9). Resveratrol has also been shown to decrease MMP-2 activity, resulting in a substantial reduction in endometrial invasiveness. Epigallocatechin gallate (EGCG) complements these effects by inhibiting VEGF-mediated angiogenesis and reducing endometrioma size (16). Polyphenols—including curcumin, resveratrol, quercetin, apigenin, and puerarin—consistently modulate estrogen signaling, inflammatory cytokines, angiogenic mediators, MMP activity, and apoptotic pathways, supporting their role as adjunctive dietary components in EMS management. However, most evidence supporting these compounds derives from *in vitro* studies, animal models, or small pilot clinical trials, and larger randomized controlled studies are needed before definitive therapeutic recommendations can be established.

### Phytoestrogens

Phytoestrogens influence EMS primarily through modulation of estrogen-dependent signaling and downstream inflammatory pathways. Pueraria flower extract reduces MMP-2 and MMP-9 expression, thereby limiting cellular adhesion and migration (18). Apigenin promotes apoptosis through the induction of BAX and BAK while suppressing MMP expression and cellular proliferation (9). Dietary isoflavone aglycones (DRIAs) have demonstrated reductions in IL-6, IL-8, COX-2, PGE2, and TNF-*α* via inhibition of NF-κB complex formation (18). These findings further support the concept that estrogen signaling and immune-mediated inflammatory pathways function in a coordinated manner in EMS pathophysiology.

### Vitamins and minerals

Antioxidant micronutrients play a key role in mitigating oxidative stress associated with EMS. Vitamins B6, A, C, and E suppress endometrial cell survival by modulating inflammatory cytokine production and enhancing macrophage-mediated immune responses (19, 20). Increased intake of these vitamins is associated with lower oxidative stress markers and improved antioxidant enzyme activity.

Observational studies consistently report lower intake of vitamins A, C, and E, as well as zinc and copper, among women with EMS compared with controls (17). Vitamins C and E are particularly relevant due to their ability to inhibit lipid peroxidation driven by reactive oxygen species. These vitamins also reduce VEGF gene expression and enhance peritoneal macrophage activity, contributing to reduced systemic oxidative stress (19).

Trace minerals such as selenium, zinc, magnesium, and iron further support antioxidant defenses through increased glutathione peroxidase and SOD activity. Magnesium and phosphorus intake demonstrate an inverse association with EMS risk, although evidence suggests whole-food dietary patterns may be more protective than isolated supplementation (17).

### Vitamin D

Vitamin D3 exhibits immunomodulatory effects by suppressing T-helper 1 (Th1) cell proliferation and reducing production of IL-2 and interferon-*γ*. Decreases in IL-6 and IL-8 have also been observed (16). In small, randomized placebo-controlled trials, vitamin D supplementation has been associated with modest but statistically significant reductions in pelvic pain (21). Epidemiologic data further supports an inverse association between serum vitamin D levels and EMS risk (22).

### Dairy

Some observational studies have reported inverse associations regarding dairy intake. Particularly calcium-rich and low-fat products have been inversely associated with systemic inflammatory stress. Higher consumption is linked to reduced TNF-*α* and IL-6 levels and a lower likelihood of EMS diagnosis (22–24). Each additional daily serving of dairy appears to confer incremental risk reduction.

### Fats and fatty acids

Dietary fat composition plays a clinically significant role in inflammatory signaling relevant to EMS. Omega-6 fatty acids serve as precursors to pro-inflammatory prostaglandins, whereas omega-3 polyunsaturated fatty acids (PUFAs) are metabolized into eicosapentaenoic acid (EPA) and docosahexaenoic acid (DHA), which are associated with reduced inflammation and pain (25). Higher intake of long-chain omega-3 fatty acids is associated with a reduced risk of EMS, while trans fatty acids and high total fat intake correlate with elevated inflammatory markers and increased disease risk (26).

However, findings across studies have not been entirely consistent. Variability in dietary assessment methods, omega-3 dosing, background dietary patterns, genetic factors, and disease severity may contribute to heterogeneous results. Some observational studies have demonstrated stronger associations than interventional trials, highlighting the need for larger, standardized randomized studies evaluating both dietary intake and supplementation strategies. Furthermore, the balance between omega-3 and omega-6 fatty acid intake may be more clinically relevant than absolute intake alone.

### Fruits and vegetables versus meat

Higher intake of fruits and vegetables is associated with reductions in oxidative stress markers and EMS risk (17). Green vegetables and fresh fruits provide folate, methionine, and vitamin B6, which support pathways involved in oxidative stress regulation and genomic stability (19, 20). High-fiber, plant-based dietary patterns enhance estrogen excretion and increase sex hormone–binding globulin levels, reducing circulating bioavailable estrogen and prostaglandin production (24).

Conversely, increased consumption of red and processed meats has been positively associated with laparoscopically confirmed EMS (19, 20).

### Spices and herbs

Bioactive compounds such as capsaicin, gingerol, and curcumin inhibit cyclooxygenase-mediated inflammatory pathways. Herbs, including oregano, rosemary, thyme, and parsley, are rich in polyphenols and contribute additional anti-inflammatory effects (24).

### Gluten and high glycemic index foods

Limited studies have suggested potential symptom improvement where adoption of a gluten-free diet has been associated with reductions in EMS-related pain, potentially through attenuation of gluten-mediated cytokine signaling and prostaglandin synthesis (27). Diets characterized by a high glycemic index are associated with increased EMS risk, suggesting glycemic control as a modifiable dietary factor (28). Dietary patterns emphasizing anti-inflammatory and antioxidant-rich foods—particularly polyphenols, omega-3 fatty acids, fruits, vegetables, adequate micronutrient intake, and glycemic control—may favorably influence molecular drivers of EMS and serve as complementary strategies alongside established medical and surgical therapies (Tables 1, 2).

**Table 1:** Dietary components relevant to EMS management.

Nutrient/food	Mechanism	Evidence type	Clinical takeaway
Omega-3 PUFA	↓ PGE2, ↓ TNF-*α*	Observational + RCT	Increased omega-3 intake may support anti-inflammatory dietary patterns.
Curcumin	↓ NF-κB, ↓ MMPs	In vitro + small trials	Potential adjunctive strategy; clinical evidence remains limited
Vitamin D	↓ IL-6, ↓ pain	RCT	Correction of deficiency may support symptom management
Red meat	↑ inflammation	Observational	Limit intake

**Table 2:** Summary of evidence supporting dietary interventions in endometriosis.

Dietary component	Proposed mechanism	Evidence type	Representative findings	Strength of evidence
Curcumin	↓ NF-κB, ↓ COX-2, ↓ IL-6	In vitro, animal, small clinical studies	Reduced inflammatory signaling and pain markers	Moderate
Omega-3 PUFAs	↓ PGE2, ↓ TNF-α	Observational + limited clinical studies	Associated with reduced pain/inflammation	Moderate
Vitamin D	↓ IL-6, immune modulation	RCT + observational	Modest reduction in pelvic pain	Moderate
Resveratrol	↓ VEGF, ↓ MMPs	Mostly preclinical	Anti-angiogenic and anti-inflammatory effects	Low–Moderate
Fruits/vegetables	Antioxidant effects	Observational	Lower EMS risk association	Moderate
Red meat/trans fats	↑ inflammatory mediators	Observational	Increased EMS risk association	Moderate

## Discussion

Plausible evidence suggests that certain dietary components can either reduce or exacerbate inflammation associated with EMS symptoms and progression. Understanding these dietary components and their food sources can help PCPs adopt a more holistic and accessible approach to EMS management. Importantly, many of the dietary mechanisms discussed in this review likely influence endometriosis through coordinated effects on both endocrine and immune pathways, rather than through isolated anti-inflammatory or hormonal actions alone.

Nevertheless, it is important to emphasize that much of the current evidence base remains observational or preclinical in nature. While emerging clinical studies provide encouraging findings for selected dietary interventions, definitive cause-and-effect relationships and standardized therapeutic dietary recommendations for EMS have not yet been established. Accordingly, dietary modification should currently be interpreted as a supportive adjunct to evidence-based medical and surgical management rather than a validated standalone treatment strategy.

Polyphenols and phytoestrogens have demonstrated several favorable biological properties, including anti-inflammatory, antioxidant, anti-proliferative, anti-angiogenic, and pro-apoptotic effects, suggesting potential utility in the prevention and modulation of EMS. Polyphenol-rich foods include herbs such as oregano, rosemary, thyme, and parsley, while phytoestrogens are commonly found in soy products, legumes, fruits, and vegetables (29). Resveratrol, a well-studied polyphenol, is present in grapes, red wine, berries, and nuts and has been associated with reduced inflammatory signaling (24). Antioxidant vitamins, particularly vitamins C and E, may further mitigate oxidative stress implicated in EMS pathophysiology. Vitamin C is abundant in fruits and vegetables such as citrus fruits, strawberries, black currants, parsley, peppers, and rosehips, whereas vitamin E is primarily obtained from nuts, seeds, vegetable oils, cereals, and green leafy vegetables.

Dietary fat composition also appears relevant to inflammatory regulation. Green leafy vegetables, nuts, soybeans, flaxseed, chia seeds, vegetable oils, and fish oils are important sources of polyunsaturated fatty acids (PUFAs), particularly omega-3 fatty acids, which have anti-inflammatory effects. In contrast, diets high in omega-6 fatty acids and saturated fats—commonly found in butter, lard, red meat, cheese, full-fat dairy products, and certain plant-based fats such as palm and coconut oils—may promote inflammation and are generally recommended to be limited in individuals with EMS (16). Despite growing evidence supporting the role of these nutrients, optimal dosing and long-term safety parameters have not yet been clearly established.

Several bioactive compounds discussed in the literature have also been evaluated within defined supplemental or dietary intake ranges, although optimal therapeutic dosing remain uncertain. Small clinical and preclinical studies have utilized curcumin supplementation commonly ranging from approximately 500–2,000 mg/day, resveratrol doses ranging from 30 to 400 mg/day, and omega-3 fatty acid supplementation providing approximately 1–3 g/day of combined EPA and DHA (30). Vitamin D supplementation strategies have varied considerably depending on baseline deficiency status and study design, with many trials utilizing daily doses between 1,000–4,000 IU/day (31). Dietary intake approaches rather than isolated supplementation has also been emphasized, including increased consumption of fatty fish, fruits, vegetables, legumes, nuts, seeds, and polyphenol-rich herbs. However, substantial variability in formulation, bioavailability, absorption, and study methodology currently limits the establishment of standardized intake recommendations for EMS management.

Although no single dietary regimen has been definitively validated as an “endometriosis diet,” emerging research suggests that structured dietary patterns may help alleviate symptoms and improve quality of life. Following diagnosis, 66% of individuals with EMS report modifying their dietary habits to reduce symptom burden and improve overall health (32). Alternative dietary patterns, such as the Mediterranean and plant-based diets, align with current research on EMS management. The Mediterranean diet—rich in fruits, vegetables, legumes, seeds, nuts, fish, and moderate dairy—incorporates minimal red meat and wine. Importantly, many proposed anti-inflammatory dietary strategies in EMS are supported not only by emerging endometriosis-specific studies, but also by broader evidence from other chronic inflammatory and metabolic conditions—including cardiovascular disease, rheumatoid arthritis, inflammatory bowel disease, and metabolic syndrome—where similar inflammatory pathways are implicated (33). Its anti-inflammatory properties may benefit individuals with EMS, as well as those with cardiovascular disease and cancer.

Similar anti-inflammatory dietary patterns have also been associated with reductions in circulating inflammatory mediators such as C-reactive protein (CRP), IL-6, and TNF-*α* in conditions including rheumatoid arthritis, inflammatory bowel disease, obesity, and metabolic syndrome, further supporting the biologic plausibility of comparable dietary mechanisms in EMS (34).

Vegetarian and vegan dietary patterns, which exclude meat and animal fats, have also been associated with increased circulating levels of sex hormone–binding globulin (SHBG). Elevated SHBG reduces bioavailable estrogen by limiting its interaction with estrogen receptors, potentially decreasing endometrial stimulation and the proliferation of prostaglandin-producing tissues (24). While further research is needed to clarify causal relationships and refine dietary recommendations, therapeutic dietary modification represents a promising, low-risk self-management strategy that may complement medical treatment and enhance symptom control and quality of life in individuals with EMS.

Emerging evidence also suggests that gut microbiota composition and estrobolome activity may influence circulating estrogen levels, inflammatory signaling, and immune regulation in endometriosis (35, 36). Dietary patterns rich in fiber and polyphenols may favorably modulate microbial diversity and estrogen metabolism, although this area remains incompletely understood and warrants further investigation.

Importantly, current evidence supports dietary modification as an adjunctive strategy rather than a replacement for established medical or surgical management.

### Clinical translation and practical considerations

Dietary strategies should be considered an adjunct to, not a replacement for, standard medical and surgical management of endometriosis. Current evidence supports a supportive role for nutrition in modulating inflammation and symptoms, but it is not sufficient to justify standalone dietary treatment. Given the heterogeneity of disease presentation, dietary recommendations should be individualized and tailored to patient preferences, comorbidities, and access to resources to improve feasibility and adherence (37). Sustainability is essential, as long-term adherence to dietary change is more important than short-term restrictive interventions. At present, there is insufficient evidence to define precise dosing, intake thresholds, or standardized dietary prescriptions for most nutrients and foods discussed. Accordingly, clinical guidance should emphasize flexible, whole-diet patterns rather than specific quantitative targets.

Given the established association between endometriosis and infertility, dietary modulation of inflammation and oxidative stress may also have implications for reproductive outcomes, including oocyte quality, implantation environment, and assisted reproductive success (38). However, current evidence remains limited and further investigation is needed.

### Dietary patterns and whole-diet approaches

Although much of the current literature focuses on isolated nutrients and bioactive compounds, growing evidence suggests that overall dietary patterns may exert a greater cumulative influence on inflammatory and metabolic pathways implicated in endometriosis. In this context, the Mediterranean diet has received the most consistent support as a potentially beneficial pattern due to its emphasis on fruits, vegetables, whole grains, legumes, nuts, fish, and olive oil, alongside lower intake of red and processed meats (38). This dietary pattern has been associated with reduced systemic inflammation and improved cardiometabolic profiles, which may indirectly influence symptom burden in endometriosis through shared inflammatory pathways.

Plant-based dietary patterns, including vegetarian and vegan diets, similarly emphasize high intake of fiber-rich foods, phytonutrients, and unsaturated fats, while limiting pro-inflammatory dietary components such as saturated fats and processed meats (39). These patterns may contribute to improved estrogen metabolism and increased sex hormone–binding globulin levels, potentially reducing bioavailable estrogen and downstream stimulation of endometrial lesions. However, the quality and composition of plant-based diets vary widely, and benefits are likely dependent on overall dietary quality rather than exclusion of animal products alone.

The Dietary Inflammatory Index (DII) provides an additional framework for evaluating diet as a composite exposure rather than focusing on individual nutrients (40). Higher DII scores, reflecting more pro-inflammatory dietary patterns, have been associated with worse inflammatory profiles in chronic disease states and may offer a useful tool for future research in endometriosis populations. In contrast, lower DII scores, reflecting anti-inflammatory dietary patterns, align closely with Mediterranean-style and whole-food-based diets.

Overall, these findings support a shift away from reductionist nutrient-focused approaches toward whole-diet and dietary pattern frameworks. The biological effects of diet likely reflect synergistic interactions among multiple nutrients, bioactive compounds, and food matrices rather than the impact of single dietary components in isolation. This systems-level perspective may better capture the complexity of diet–inflammation interactions relevant to endometriosis pathophysiology.

### Limitations

Several limitations should be considered when interpreting the findings of this integrative review. First, a substantial proportion of the mechanistic evidence supporting the anti-inflammatory and anti-angiogenic effects of dietary compounds—particularly polyphenols and phytoestrogens—derives from *in vitro* and animal model studies. While these models provide valuable insight into molecular pathways relevant to endometriosis, their findings may not fully translate to human physiology, clinical symptomatology, or long-term disease outcomes.

Second, randomized controlled trials (RCTs) in humans remain limited for many dietary components discussed in this review. Where clinical trials exist, they are often characterized by small sample sizes, short intervention durations, variable dosing strategies, and heterogeneous outcome measures, which constrain the strength of causal inference and limit generalizability. As a result, many dietary recommendations remain supported primarily by observational or mechanistic evidence rather than high-level clinical trial data.

Third, observational nutrition studies are inherently subject to residual confounding and measurement error, including inaccuracies in self-reported dietary intake, unmeasured lifestyle factors, and socioeconomic influences. These limitations complicate efforts to isolate the independent effects of specific dietary components on EMS risk or symptom severity.

Fourth, there is considerable variability in the bioavailability, metabolism, and dosing of dietary supplements, particularly for compounds such as curcumin, resveratrol, and quercetin. Differences in formulation, absorption, gut microbiota interactions, and individual metabolic responses may influence biological effects and clinical outcomes, limiting the ability to extrapolate standardized recommendations from existing studies.

Finally, this review focused primarily on dietary factors influencing inflammatory mechanisms of EMS and did not comprehensively address hormonal dietary interventions, gene–diet interactions, or long-term dietary adherence and safety. These areas represent important directions for future research.

Additionally, substantial heterogeneity exists among nutrition studies evaluating dietary fats and supplementation strategies, limiting direct comparison across studies and complicating interpretation of causal relationships (21). Also, the search strategy did not include certain lay or variably defined clinical terminology, such as “estrogenic dominance,” which may have limited retrieval of some relevant studies discussing hormonal influences in endometriosis.

Despite these limitations, the convergence of mechanistic, epidemiologic, and emerging clinical evidence supports a biologically plausible role for dietary modulation as an adjunctive strategy in the management of endometriosis. Future large-scale, well-designed randomized trials are needed to establish optimal dietary patterns, dosing thresholds, and long-term clinical efficacy.
